# Unraveling the Complexities of Chronic Low Back Pain: A Comprehensive Case Study

**DOI:** 10.7759/cureus.83523

**Published:** 2025-05-05

**Authors:** Waleed Sultan, Samuel Ayad, Ruth Pawlowski

**Affiliations:** 1 Family Medicine, Conemaugh Memorial Medical Center, Johnstown, USA; 2 Medicine, Beni Suef University Faculty of Medicine, Beni Suef, EGY

**Keywords:** axial back pain, back pain, chronic low back pain (clbp), lower back pain (lbp), radicular back pain, spinal cord stimulator

## Abstract

Chronic low back pain (LBP) is a growing global epidemic and a leading cause of disability worldwide. The distribution of pain, whether axial, radicular, or mixed, and its characteristics can point to the underlying pathology. Physical rehabilitation, pharmacological interventions, psychological support, innovative therapies, and surgical interventions are common approaches in LBP management. This comprehensive case study explores a complex case of chronic LBP, investigating multiple underlying pathologies and the challenges in finding a sustainable management approach. This report highlights the necessity of a multidisciplinary approach to managing chronic LBP. Understanding the complexities of chronic LBP is crucial in developing personalized and comprehensive management strategies.

## Introduction

Low back pain (LBP) has emerged as a growing global epidemic, impacting nearly 10% of the world's population. Despite being a leading cause of disability worldwide with an enormous economic burden, it has not received proportional attention [1]. Chronic LBP usually persists for over 4-12 weeks or recurs after acute presentation, manifesting in axial, radicular, or mixed forms. This clinical distinction offers insights into the underlying pathology. Axial LBP can stem from diverse sources, including paraspinal muscles, intervertebral discs, facet joints, and sacroiliac joints.

Combination pharmacotherapy and physical rehabilitation are common primary methods, but often lack sustained positive outcomes. Interventions like transforaminal epidural steroid injections, radiofrequency ablations, neurostimulation, and spinal surgical procedures find widespread use in selected cases [2]. The World Health Organization (WHO) issued its inaugural guidelines for the non-surgical management of chronic primary LBP in 2023 [3]. However, there is also a growing concern regarding the potential overtreatment of chronic LBP [4]. Recent advancements, such as pain reprocessing therapy, focus on psychological factors, including the patient's beliefs, perceptions, and expectations, showing promise [5]. This study delves into a case of chronic LBP with multiple underlying pathologies refractory to various therapies and interventions.

## Case presentation

A 59-year-old female with a medical history of type 2 diabetes mellitus, obesity (BMI: 36 kg/m²), obstructive sleep apnea, chronic obstructive pulmonary disease, and a history of triple-vessel coronary artery bypass graft surgery for coronary artery disease presented with chronic LBP persisting for over 16 years. The pain was described as sharp and radiating to both hips, with associated tingling and numbness in the right thigh and knee. The intensity of the pain fluctuated between 2 and 9 on a scale of 10, worsening with standing and walking but relieved by sitting, lying down, and opioid use. Notably, the patient denied any weakness, incontinence, or saddle anesthesia.

**Figure 1:**
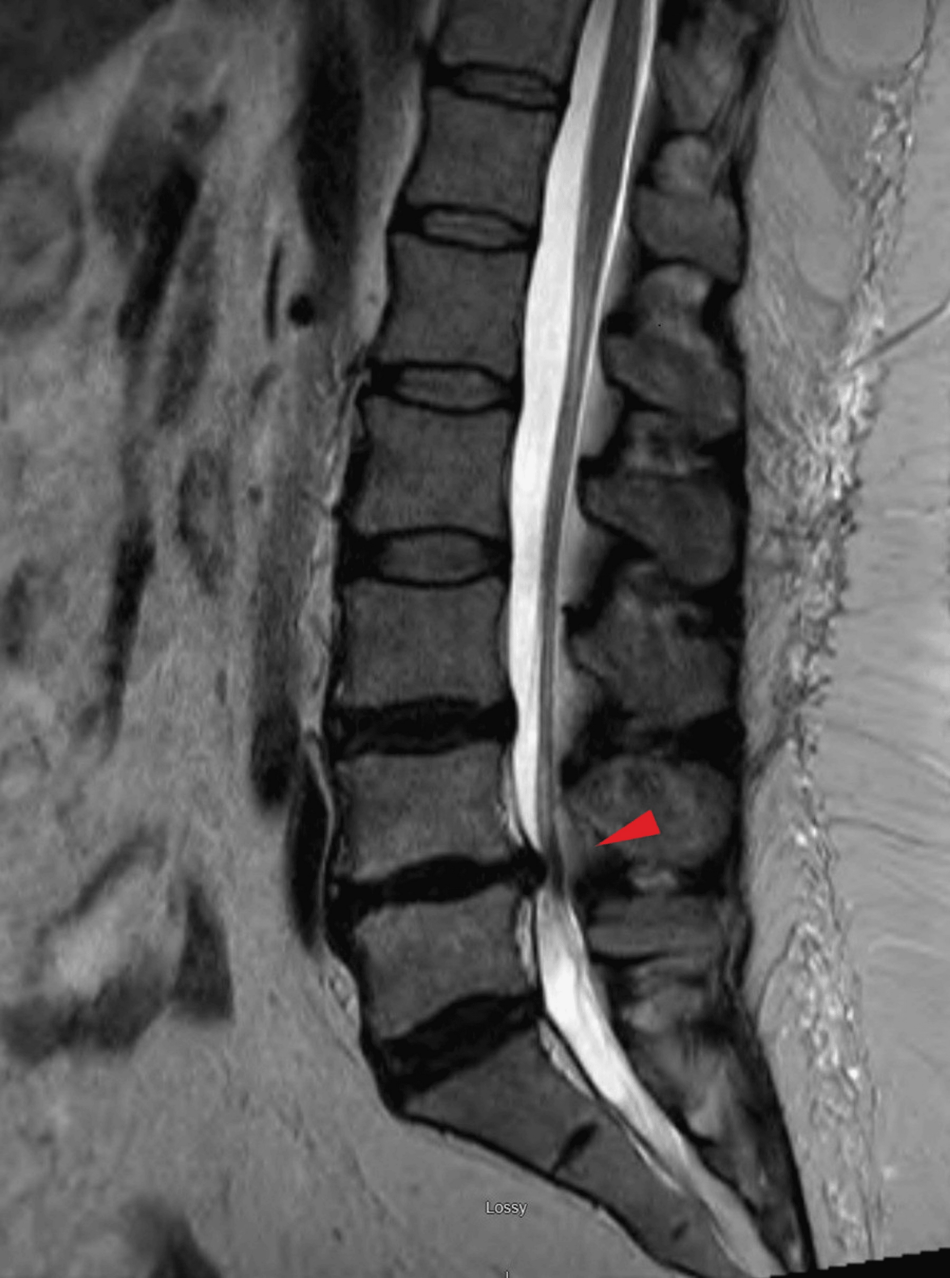
MRI of the lumbar spine shows L4-5 degenerative disc disease and diffuse annular bulging: degenerative facet, flaval ligament hypertrophy, severe central canal compromise (Age: 49 years).

**Figure 2:**
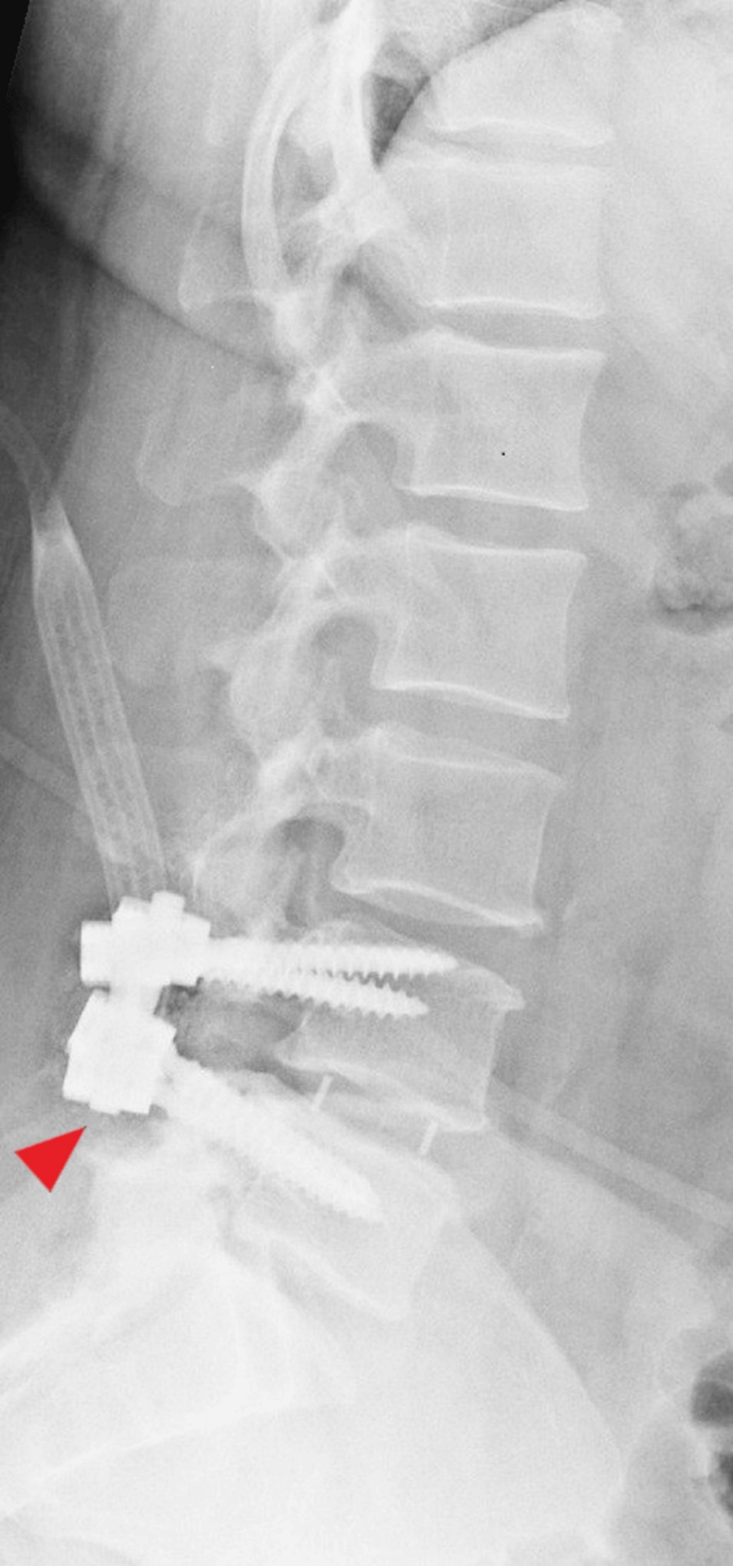
Postoperative X-ray of lumbar spine demonstrating posterior fusion hardware at L4-L5 with bilateral transpedicular screws with discectomy (Age: 49 years).

The symptoms began gradually at age 43 without any traumatic event. At that time, an initial lumbar spine MRI revealed degenerative changes and disc protrusions at the L3-4 and L5-S1 levels. Despite a range of interventions that were introduced subsequently and additionally, including opioids, non-steroidal anti-inflammatory drugs (NSAIDs), muscle relaxants, serotonin and norepinephrine reuptake inhibitors (SNRIs), physical therapy, and nerve blocks, the patient did not experience sustained pain relief. At age 49, a follow-up MRI showed the resolution of the disc issues at L3-4 and L5-S1. Still, new findings emerged at L4-L5, including severe central canal compromise, as shown in Figure 1. Surgical intervention included a decompressive laminectomy and transforaminal lumbar interbody fusion (TLIF) with fixation, as shown in Figure 2, but postoperatively, pain persisted despite continued physical therapy and pharmacotherapy. A subsequent MRI six months later demonstrated degenerative changes at L3-4 with mild central canal compromise.

**Figure 3:**
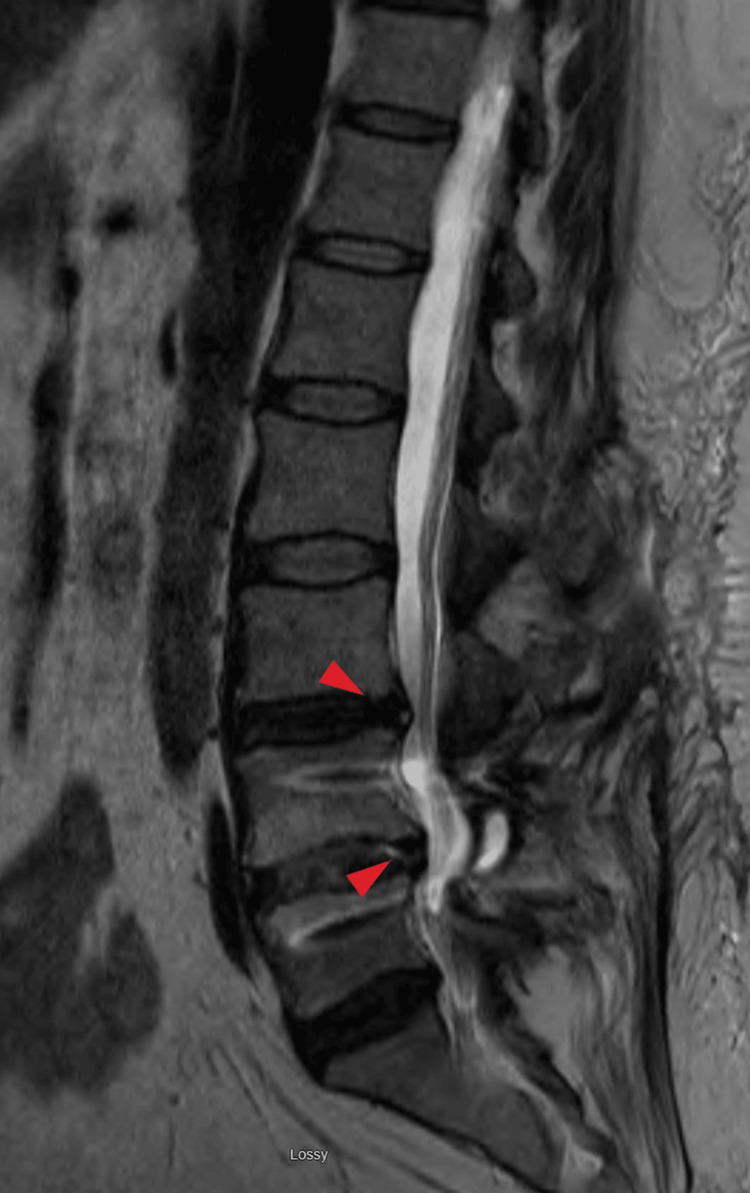
MRI of the lumbar spine shows interval postoperative changes at the L4-5 level, and degenerative changes at L3-4 causing mild central canal compromise (Age: 55 years).

**Figure 4:**
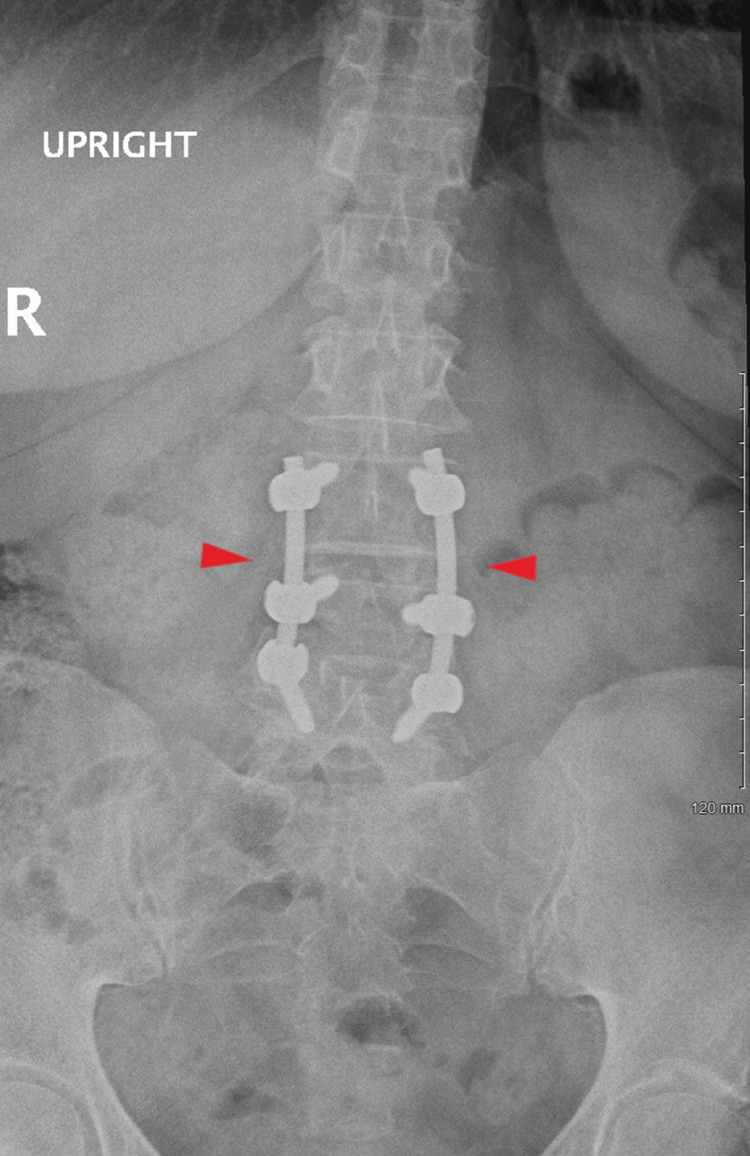
Lumbar X-ray showing postoperative changes of the posterior spinal fixation and decompression from L3-L5 with good alignment and good placement of hardware (Age: 56 years).

At age 50, the patient underwent epidural steroid injections, but these provided minimal relief. She declined a spinal cord stimulator (SCS) implantation. Despite ongoing pharmacological management with opioids and NSAIDs, symptoms persisted with moderate disability according to the Oswestry Disability Index (ODI). An MRI at age 55 revealed spinal stenosis at L3-L4, as shown in Figure 3. Surgery at age 56 included hardware revision, decompression, and fusion at the L3-L5 levels, as shown in Figure 4, but again, the pain remained unresolved.

The patient continued to experience refractory pain despite sacroiliac joint injections and ongoing pharmacotherapy. After a trial of SCS with 60% pain relief, a permanent stimulator was implanted. However, within six months, pain recurrence led to multiple adjustments of the stimulator. Adjuvant therapy with topiramate and zonisamide was introduced, but no significant improvement was noted. At age 58, the patient requested the removal of the stimulator due to its inefficacy, and the procedure was completed without complications. Despite this, her symptoms persisted. A diagnostic block provided brief pain relief, and pharmacological management continues as the patient explores additional treatment options, including caudal epidural steroid injections. Table 1 summarizes the symptoms, progression, and interventions provided.

**Table 1:** Structured timeline of symptoms, imaging findings, and interventions LBP: low back pain; TLIF: transforaminal lumbar interbody fusion; PT: physical therapy; SCS: spinal cord stimulator; ESI: epidural steroid injection

Age (years)	Symptoms	Imaging: Finding	Intervention
43	Gradual onset of LBP, radiating to the hips	MRI: L3-4 & L5-S1 disc protrusions	Conservative management (medications, PT, blocks)
49	Persistent pain	MRI: L4-5 severe central canal compromise	TLIF at L4-5
50	Persistent pain, right thigh/knee numbness	MRI: L3-4 mild central canal compromise	Epidural injections; refused SCS
55	Worsened pain	MRI: L3-4 spinal stenosis	Considered for surgery
56	Persistent pain	MRI: postoperative changes, degenerative at L3-4	Hardware revision and fusion at L3-L5
57	Temporary pain relief	X-Ray: confirmed proper stimulator placement	SCS trial and permanent implant
58	Pain recurrence	No imaging	SCS removal, diagnostic block
59	Persistent chronic pain	No imaging	Pharmacologic management, caudal ESI

## Discussion

Epidemiology and classification

LBP affected approximately 619 million individuals in 2020, with projections reaching 843 million by 2050. It is the leading cause of disability worldwide, contributing to 7.7% of all Years Lived with Disability (YLDs) [1].

Pain is typically classified as nociceptive (somatic or visceral origin) or neuropathic (originating from the somatosensory system). LBP may be categorized by distribution: axial pain, which is localized to the lower back, or radicular pain, which radiates into the lower extremities. Many patients present with a combination of both types. Axial LBP commonly arises from paraspinal musculature, intervertebral disc disruption (IDD), facet joint pain (FJP), or sacroiliac joint pain (SIJP). In contrast, radicular pain is most frequently associated with herniated intervertebral discs (HID) or foraminal stenosis [2,6]. A summary of LBP types, sources, physical findings, and characteristics is presented in the diagram in Table 2 [2].

**Table 2:** Chronic low back pain: types and common causes and characteristics Reference: Hooten and Cohen, 2015 [2]

Type	Type	Features
Axial	Muscular/Ligamentous	Delayed onset, localized lumbosacral pain, reduced mobility, muscle tenderness and spasms, may follow trauma.
	Sacroiliac Joint Pain	Axial below L5; buttock/thigh radiation worsens with sitting/standing; tenderness on exam; L5/S1 nerve involvement.
	Intervertebral Disc Disruption	Common in ages under 45 years; worsens with loading and sitting; midline tenderness; improves with rest.
	Facet Joint Pain	Common in ages over 65 years; worsens with standing; improves with sitting; pelvic tilt pain.
Axial and Radicular	Spinal Stenosis	Age usually over 65 years; canal narrowing and spondylosis; radicular pain in legs; improves with lumbar flexion.
Radicular	Herniated Disc	Gradual onset in young adults; sharp, burning pain; worsens with coughing/sneezing/flexion; dermatomal distribution.

Identifying non-musculoskeletal causes of LBP, including neoplastic, inflammatory, visceral, infectious, vascular, endocrine, and traumatic etiologies, is essential. These are typically screened using “red flag” indicators, incorporating patient history, risk factors, and physical exam findings [6].

Diagnostic approach

Despite recommendations against routine imaging, modalities such as lumbar radiography, CT, and MRI are commonly used. However, the American College of Physicians (ACP) and the American Pain Society (APS) advise reserving imaging for patients being considered for surgery or when serious underlying conditions are suspected [7]. Electrodiagnostic tests such as electromyography and nerve conduction studies are particularly useful in evaluating radicular pain and lumbar spinal stenosis. Diagnostic injections of facet joint blocks, selective nerve root blocks, and sacroiliac joints can assist in confirming the pain generator and guiding therapeutic decisions, including suitability for interventional treatments like percutaneous radiofrequency denervation.

Management strategies

Pharmacologic treatments for chronic LBP include NSAIDs, muscle relaxants, antidepressants, opioids, and adjuvant medications. However, many of these lack robust long-term evidence and are associated with notable side effects. Gabapentinoids and topiramate, used for radicular pain, demonstrate inconsistent efficacy. Intradiscal steroid injections have generally shown disappointing results in discogenic axial LBP [8]. Epidural steroid injections offer short-term relief but come with rare yet significant risks. Biologic agents, such as epidural etanercept, have yielded mixed outcomes. Non-pharmacological therapies such as exercise, spinal manipulation, acupuncture, and massage may relieve short-term symptoms. However, their long-term effectiveness remains inconclusive. Psychologically informed treatments, including cognitive-behavioral therapy (CBT), have positively impacted emotional functioning. Mindfulness-based stress reduction techniques may aid in pain acceptance, though evidence is variable. In 2023, the WHO released its first guidelines on the non-surgical management of chronic primary LBP, emphasizing integrated, patient-centered care approaches summarized in Table 3 [3].

**Table 3:** Summary of the WHO Guidelines for non-surgical treatment of low back pain Reference: WHO Guideline for Non-surgical Management of Chronic Primary Low Back Pain in Adults in Primary and Community Care Settings, 2023 [3] NSAIDs: non-steroidal anti-inflammatory drugs

Conditional recommendation in favor of use	Conditional recommendation against use		No recommendations/No intervention/inadequate evidence
Moderate certainty: NSAIDs.		Moderate certainty: Opioids.	-Respondent therapy. - Cognitive therapy. -Mindfulness-based stress reduction therapy. -Acetaminophen. -Benzodiazepines. - Cannabis-related preparations. -Topical Brazilian arnica Ginger. -Topical white lily. -Topical combination herbal transdermal compress. -Non-pharmacological weight loss.
Low certainty: Structured learning. -Structured exercise therapy. -Needling therapy, e.g., acupuncture. -Topical Cayenne pepper. -Multicomponent biopsychosocial care.		Low certainty: Therapeutic ultrasound. -Selective norepinephrine reuptake inhibitor (SNRI) antidepressants.	
Very low certainty: Spinal manipulation. -Massage. -Operant therapy. -Cognitive behavioral therapy.		Very low certainty: Traction. -Transcutaneous electrical nerve stimulation. -Tricyclic antidepressants. -Nerve stimulation. -Lumbar braces, belts, and/or supports. -Tricyclic antidepressants, anticonvulsants, muscle relaxants, glucocorticoids, injectable local anesthetics. -Devil's claw (Harpagophytum procumbens), white willow (Salix spp.). -Pharmacological Weight Loss.	
Good practice: Mobility assistive products.			

Surgical options for LBP include decompression procedures (e.g., laminectomy), discectomy, foraminotomy, disc arthroplasty, and spinal fusion. Among these, decompressive laminectomy has shown efficacy for lumbar spinal stenosis [9]. Advanced neuromodulation strategies, including spinal cord stimulation (SCS), dorsal root ganglion stimulation, motor cortex stimulation, and deep brain stimulation (DBS), offer promising results for refractory cases, particularly high-frequency SCS in chronic radiculopathy. DBS targeting the periaqueductal gray, periventricular gray, or thalamic regions has shown potential in treating failed back surgery syndrome and remains an area of future research [10]. Intrathecal drug delivery systems are reserved for severe, inoperable cases such as refractory pain or cancer-related LBP.

Contemporary research on chronic pain highlights its complex interaction with the brain’s emotional and motivational networks. Brain structures, including the somatosensory and insular cortices, amygdala, and nucleus accumbens, have been implicated in the transition from acute to chronic pain states. Pain reprocessing therapy (PRT) is a novel approach that frames chronic pain as a reversible, learned brain process. It aims to shift patients' perceptions and reduce fear-avoidant behaviors by reframing pain as non-threatening and brain-generated. Preliminary studies suggest that PRT may relieve chronic pain, including LBP, particularly when emotional and cognitive factors are significant contributors [5].

## Conclusions

This case highlights the complexity of managing chronic LBP in patients with multifactorial etiologies and comorbidities, despite multiple surgical and non-surgical interventions. It underscores the importance of a multidisciplinary approach, combining rehabilitation, pharmacologic management, psychological support, and interventional or surgical options tailored to individual needs. Future research should focus on predictive models for surgical and neuromodulation outcomes and standardized patient selection tools to improve treatment efficacy and reduce unnecessary procedures. Precision medicine strategies incorporating clinical, imaging, and psychosocial factors hold promise for optimizing care in chronic LBP.

## References

[1] (2023). Global, regional, and national burden of low back pain, 1990-2020, its attributable risk factors, and projections to 2050: a systematic analysis of the Global Burden of Disease Study 2021. Lancet Rheumatol.

[2] Hooten WM, Cohen SP (2015). Evaluation and treatment of low back pain: a clinically focused review for primary care specialists. Mayo Clin Proc.

[3] (2023). World Health Organization. WHO guidelines for non-surgical management of chronic primary low back pain in adults in primary and community care settings. Published December 14. WHO Guideline for Non-surgical Management of Chronic Primary Low Back Pain in Adults in Primary and Community Care Settings.

[4] Deyo RA, Mirza SK, Turner JA, Martin BI (2009). Overtreating chronic back pain: time to back off?. J Am Board Fam Med.

[5] Ashar YK, Gordon A, Schubiner H (2022). Effect of pain reprocessing therapy vs placebo and usual care for patients with chronic back pain: a randomized clinical trial. JAMA Psychiatry.

[6] DePalma MJ, Ketchum JM, Saullo T (2011). What is the source of chronic low back pain and does age play a role?. Pain Med.

[7] Chou R, Qaseem A, Owens DK, Shekelle P (2011). Diagnostic imaging for low back pain: advice for high-value health care from the American College of Physicians. Ann Intern Med.

[8] Kuijpers T, van Middelkoop M, Rubinstein SM, Ostelo R, Verhagen A, Koes BW, van Tulder MW (2011). A systematic review on the effectiveness of pharmacological interventions for chronic non-specific low-back pain. Eur Spine J.

[9] Pannell WC, Savin DD, Scott TP, Wang JC, Daubs MD (2015). Trends in the surgical treatment of lumbar spine disease in the United States. Spine J.

[10] Farrell SM, Green A, Aziz T (2018). The current state of deep brain stimulation for chronic pain and its context in other forms of neuromodulation. Brain Sci.

